# Pre- and postoperative angle kappa in MIOL patients after touch-up LASIK

**DOI:** 10.1371/journal.pone.0283578

**Published:** 2023-04-06

**Authors:** Amr Saad, Andreas Frings, Vasyl Druchkiv, Toam Katz

**Affiliations:** 1 Department of Ophthalmology, University Hospital Duesseldorf, Duesseldorf, Germany; 2 Department of Ophthalmology, University Medical Center Hamburg-Eppendorf, Hamburg, Germany; 3 Clinica Baviera, Valencia, Spain; 4 Care-Vision, Martinistraße, Hamburg, Germany; University of Cape Coast, GHANA

## Abstract

**Purpose:**

To study the influence of angle kappa (κ) on visual acuity after implantation of a multifocal intraocular lens (MIOL) and consecutive “touch-up” corneal refractive surgery with Laser-in-situ-Keratomileusis (LASIK).

**Methods:**

This retrospective multicenter study included patients who underwent MIOL surgery and consecutive LASIK (= Bioptics) in the period from 2016 to 2020 at Care Vision Refractive Centers in Germany. Our study was approved by the local ethics committee at the University in Duesseldorf (approval date: 23.04.2021) and conducted according to the tenets of the Declaration of Helsinki and Good Clinical Practices Guidelines. The pre- and post-operative κ of 548 eyes were measured using a Scheimpflug-based imaging system. Corrected distance visual acuity (CDVA) and the safety index (SI) were analyzed in relation with κ. For a more detailed analysis, the cohort was divided into pre-operative hyperopic and myopic patients to show group-specific differences.

**Results:**

There was a significant decrease (p<0.001) in the magnitude of κ after MIOL implantation and Bioptics. However, there was almost no significant correlation of κ on CDVA and SI, pre- and postoperatively.

**Conclusion:**

A large κ is not a significant risk factor for poor visual acuity. Therefore, it is not a suitable clinical predictor of postoperative outcomes after a Bioptic procedure.

## Introduction

With the invention of corneal tomography, the measured parameters have become much more important in refractive surgery, as they provide accurate values of corneal curvatures [1]. Angle kappa (κ) is one of these parameters, which is defined as the difference between two optical axes of the eye: the pupillary axis and visual axis. First one, the pupillary axis, passes perpendicular to the cornea and intersects the center of the entrance pupil. Second one, the visual axis, is an imaginary line that connects the fixated object and the center of the fovea. Thus, it is also called the foveal-fixation axis [2]. The chord distance between the intersection of the pupillary axis with the cornea and the corneal light reflex (vertex normal) makes the practical measurement of the angle κ as a distance between the intersections of both axes with the corneal surface. Pentacam® systems (Oculus Optikgeräte GmbH, Wetzlar, Germany) can identify the distance in the x and y directions in the first Purkinje image. This Scheimpflug-based imaging system proved to be a reliable measurement method [3].

In the last years multifocal intraocular lenses (MIOL) popularity increased strongly. With a wide range of lenses available, individual needs can be addressed and patients’ quality of life can be positively influenced [4]. In cataract surgery and in the correction of refractive errors, the importance of MIOL centration regarding the quality of visual outcomes has been discussed numerously. Because a decentration of MIOL among other reasons is associated with postoperative optic phenomena such as glare and halos, quality of life may suffer accordingly. Next to blurred vision, reduced contrast sensitivity is also a common side effect after MIOL implantation [5–7]. Angle κ might be a possible predictor of such phenomena. Some studies suggest large angle κ greater than 0.4 mm has a negative impact on the postoperative outcome [8, 9]. Velasco-Barona et al. could not prove any relationship [10].

Residual ametropia, especially for intermediate vision, is also a reason for poor patient satisfaction [9, 11, 12]. One possible approach to this problem is a touch-up procedure which is an Excimer laser enhancement after MIOL implantation. It showed reliable refractive outcomes and high patient satisfaction, because LASIK is known to be a successful procedure offering reasonably accurate results [13]. However, rainbow glare and halos are possible side effects of femtosecond laser procedure [14].

According to current research, there is little evidence of the relationship between κ and the visual outcome in MIOL patients. Besides, only few eyes were involved in most of previous studies and none of them included patients underwent a touch-up procedure. Based on the latest state of studies, it is still unclear if there is a higher risk due to large κ [10, 15].

The goal of our study is first, to examine if there is a significant change in κ pre- to postoperatively. Second, we want to determine the relationship between κ and CDVA and the safety index (SI). Our aim is to show whether angle κ is a parameter to be considered avoiding negative effects in clear lens refractive surgery (MIOL surgery). This would play an important role in patient selection.

## Materials and methods

Clear lens extractions performed in 548 presbyopic eyes (235 male and 313 female) without previous surgeries or pathologies, from 45 to 76 years of age (mean: 53.8 ± 5 years) were included in our study. The eyes (patients) were divided into pre-operative myopic and hyperopic groups. All eyes had gone through a touch-up LASIK to correct a residual ametropia. Only non-complicated eyes were included. A common complication such as secondary cataract, macular edema or severe dry eye after LASIK which reduces the corrected visual acuity below SI of 0.8, reduces the ability to focus on the tomograph and visual test target and does not allow reproducible visual acuity and angle κ values. Hence, we excluded eyes with SI < 0.8. Analyzing only eyes with good SI, excludes this bias and improves the resolution of the correlation between angle κ and SI to clinically meaningful range.

Our multicenter retrospective study included data retrieved from the Care Vision Refractive Database (R&D Department of CARE Vision company, Germany). Our study adhered to the tenets of the Declaration of Helsinki and the GDPR (General Data Protection Regulation). All patients voluntarily provided written informed consent for data analysis during the surgery recruitment process.

The implanted MIOL types were hydrophilic refractive diffractive MIOLs (PhysIOL, Liege, Belgium). Pre-operative corneal cylinder of 2 diopter or more indicated a toric MIOL (Model FineVision Pod F toric) which was implanted in 49 eyes (8.9%). In all other eyes non-toric MIOLs (FineVision Pod F in 282 eyes (51.5%) and FineVision Micro F in 217 eyes (39.6%)) were implanted. The surgical procedures were carried out according to an internal standard protocol under topical anesthesia. All implanted MIOLs were centered at the pupil center. The power of the trifocal MIOL was computed using a laser interferometer (IOL Master 500, Zeiss, Germany) and the Haigis formula.

To estimate the angle κ values, the distance between the pupilar center and corneal light reflex was determined using a Scheimpflug-based imaging system (Pentacam®, Oculus Optikgeräte GmbH, Wetzlar, Germany). The values of angle κ were presented not in degrees of angle but as κ magnitude. The offset of the pupil center image on the corneal surface from the corneal apex is given by the Pentacam as “x” and “y” values and was converted to a nasal offset in a polar (Vector) value of “κ magnitude” measured in micrometer (μ), the κ magnitude of the left eyes was converted to a standard right eye reference in which more nasal means more minus x value. Angle κ is referred as “κ”, “angle κ” or “κ magnitude” interchangeably. CDVA was measured at a viewing distance of 6 meters (20 feet) to a Snellen Visual Chart [16]. SI was calculated as the relation between the post-operative corrected distance visual acuity (CDVA) and the pre-operative CDVA [17]. Angle K, SI and CDVA were taken pre-operatively and after the final LASIK. Generally, preoperative measurements were made 4 to 6 weeks before surgery and touch-up LASIK was performed on average 3 months after MIOL surgery. No decentering of the ablation zone occurred. Preoperative angle κ was compared with the postoperative measurement at follow-up, which was performed at least 3 months after Bioptics (final treatment). Because this is a multicenter retrospective study, we only analyzed data that had been consistently and completely documented in all eyes.

The correlation between angle κ and continuous variables CDVA and SI was estimated using linear regression. A p value less than 0.05 was considered statistically significant. All analysis was performed with R [18].

## Results

The statistical analysis showed that pre- and postoperative angle κ of our cohort (548 eyes) were homogenously distributed around the mean values. It further showed that the κ values of the hyperopic eyes were larger than those of the myopic eyes.

### Refractive outcomes

**Fig 1:**
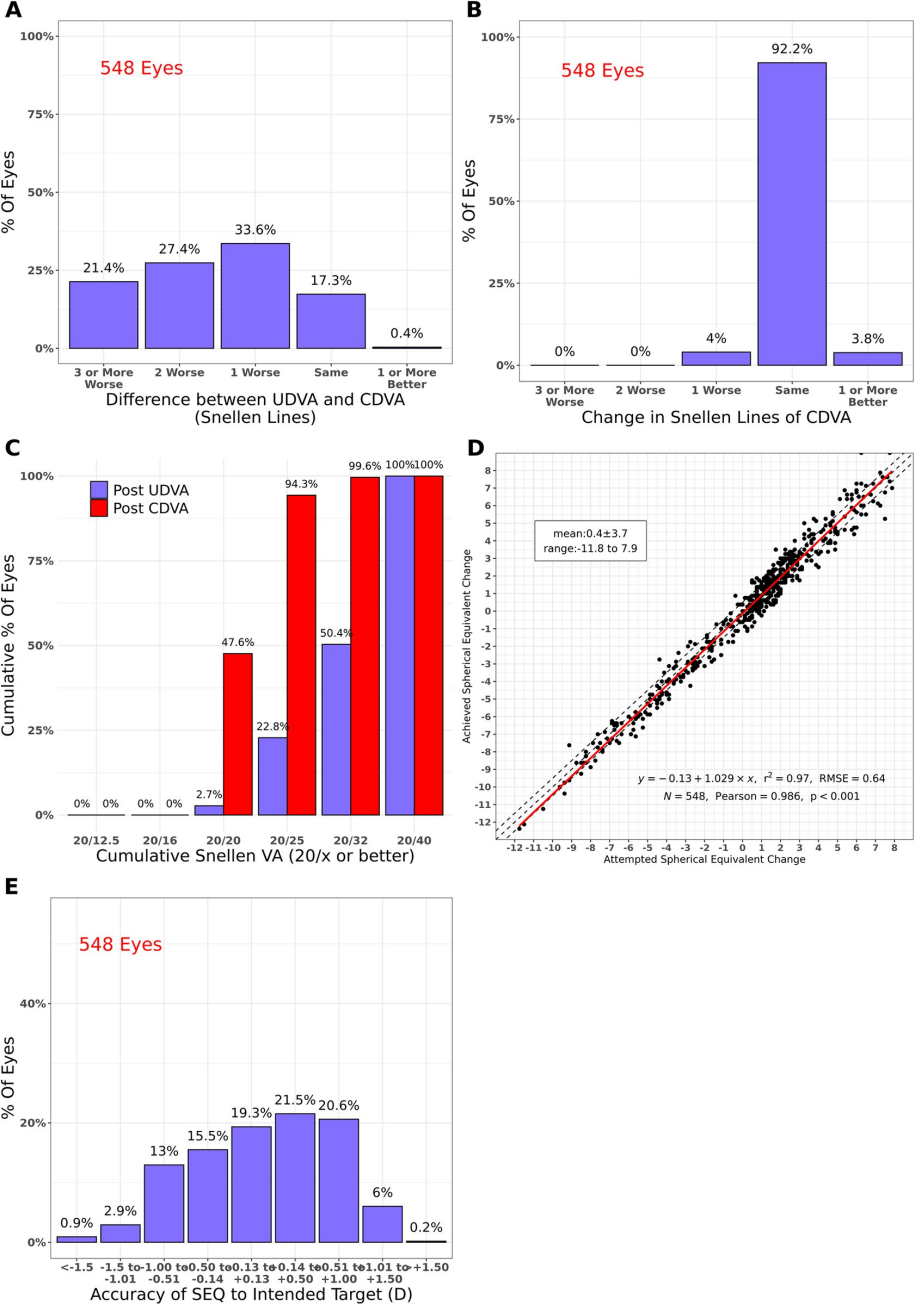
Standard graphs reporting visual outcomes: Preoperative vs postoperative (MIOL implantation). UDVA: uncorrected distance visual acuity, CDVA: corrected distance visual acuity, VA: visual acuity, SEQ: spherical equivalent, D: diopter(s), r^2^: coefficient of determination, RMSE: root mean squared error, N: number of eyes, Pearson: Pearson Correlation Coefficient.

**Fig 2:**
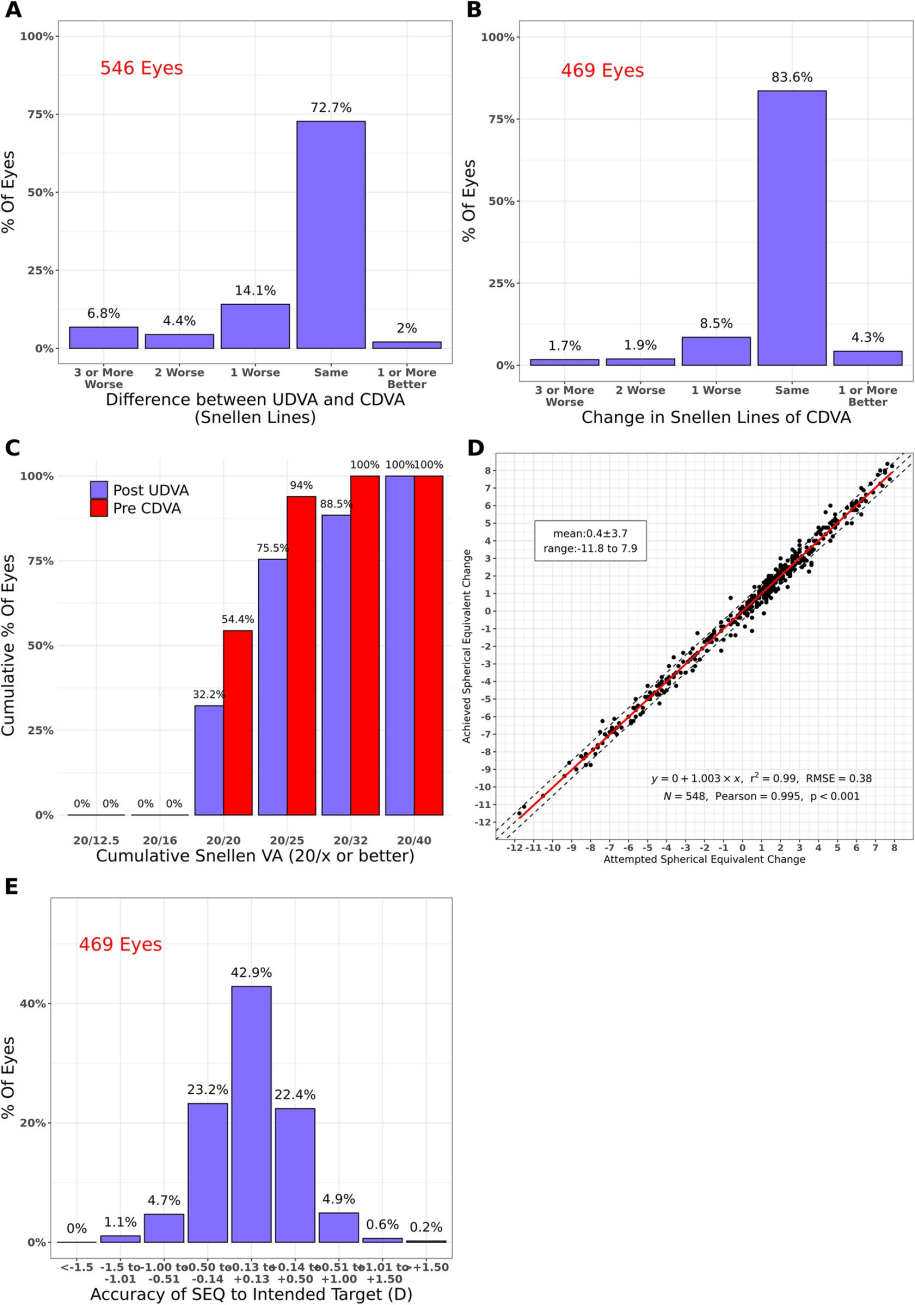
Standard graphs reporting visual outcomes: Preoperative vs postoperative (touch-up LASIK). UDVA: uncorrected distance visual acuity, CDVA: corrected distance visual acuity, VA: visual acuity, SEQ: spherical equivalent, D: diopter(s), r^2^: coefficient of determination, RMSE: root mean squared error, N: number of eyes, Pearson: Pearson Correlation Coefficient.

Since all patients received two consecutive treatments, the visual outcomes of two different intervals were examined. First, the preoperative visual acuity (VA) is compared with the postoperative VA after MIOL implantation (Fig 1). Second, preoperative VA is compared with VA after touch-up LASIK (Fig 2). The reported refractive outcomes are in accordance with the Standard Reporting in Refractive Surgery [19].

Graphs A and C respectively show the efficacy of the treatments. Graph B shows the safety, graph D the predictability as a correlation between attempted and achieved spherical equivalent (SEQ), and graph shows the accuracy of SEQ regarding the intended target.

After MIOL implantation UDVA was equal to or better than CDVA in 17.7% of eyes, while this proportion increased to 74.7% after LASIK (Figs 1A and 2A).

All eyes were within 1 Snellen Line of CDVA after MIOL implantation, but only 96.4% after LASIK (Figs 1B and 2B).

After MIOL surgery, only 2.7% of eyes had UDVA of 20/20 compared with 47.6% that had CDVA of 20/20 (Fig 1C). On the other hand, 32.2% of eyes achieved UDVA of 20/20 after LASIK compared with 54.4% CDVA in the preoperative condition (Fig 2C).

A strong correlation between attempted and achieved SEQ was observed in both intervals (Pearson = 0.986 and 0.996, p<0.001, Figs 1D and 2D).

Postoperative SEQ was within ±0.5 D of emmetropia in 56.3% of eyes after MIOL implantation and in 88.5% of eyes after LASIK (Figs 1E and 2E).

### Change of angle κ

**Fig 3:**
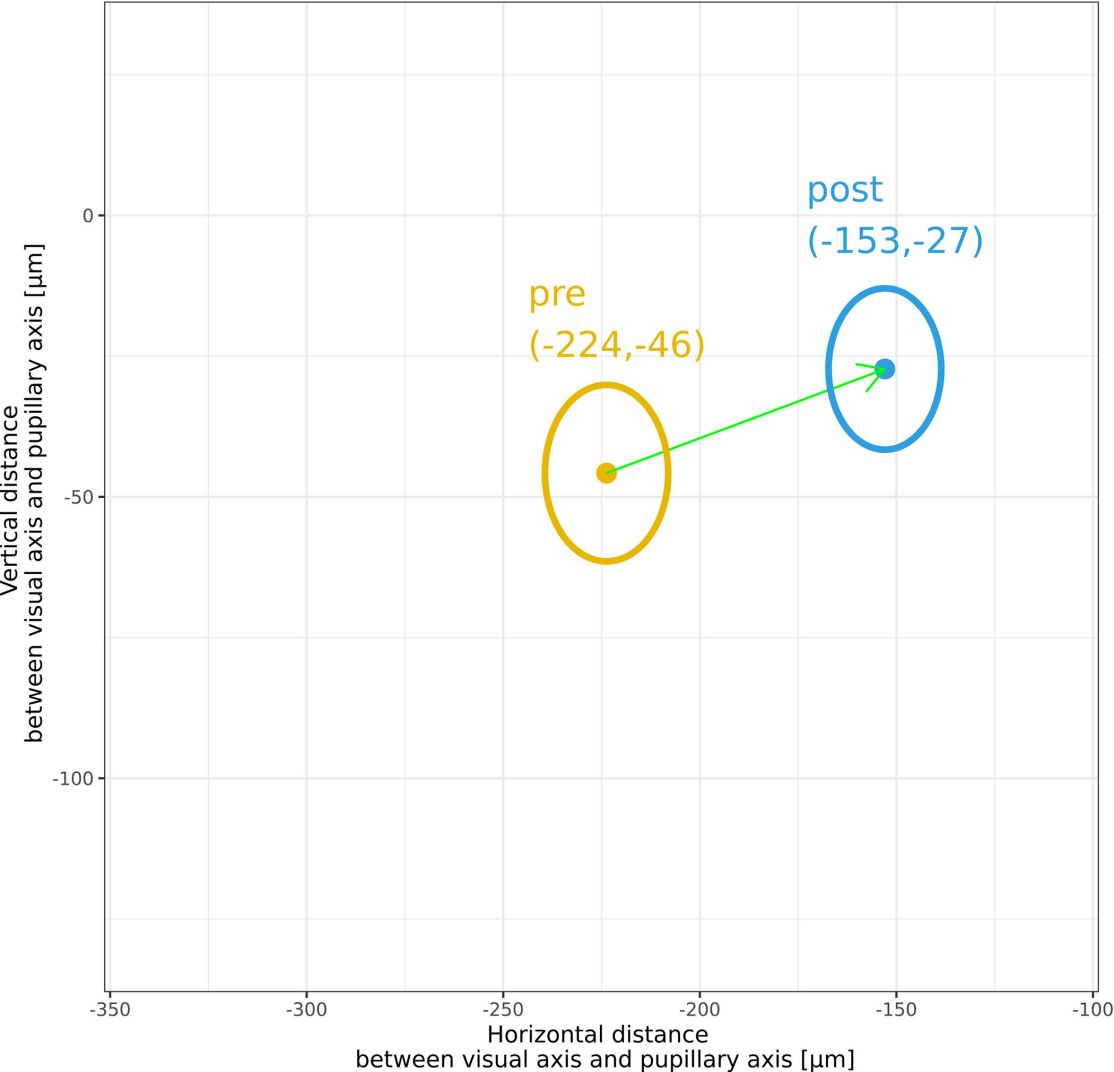
Change of angle kappa magnitudes: Difference vector in terms of preoperative and postoperative visual axis (overall group). pre: preoperative, post: postoperative.

**Fig 4:**
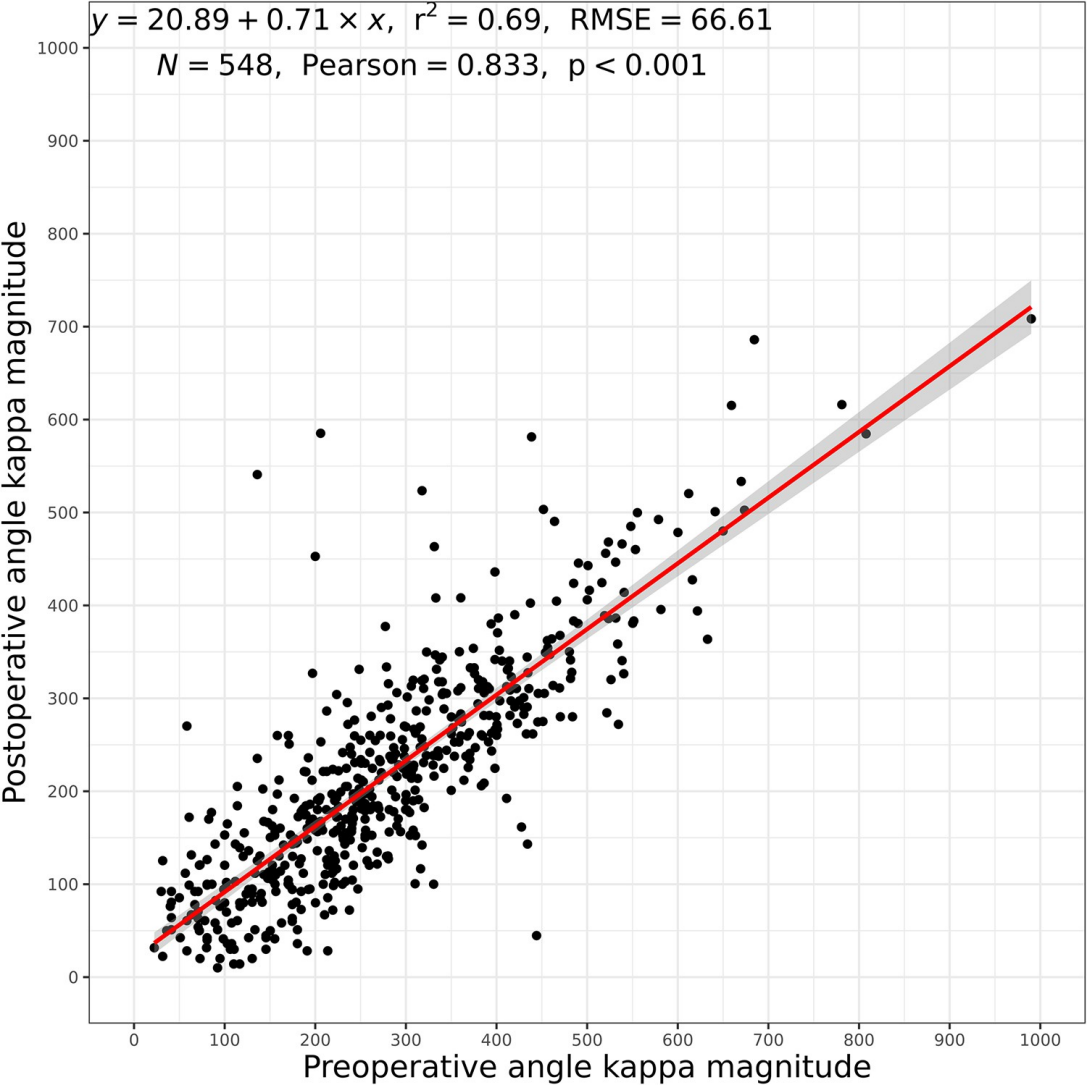
Correlation plot: Change of angle kappa magnitudes (overall group). r^2^: coefficient of determination, RMSE: root mean squared error, N: number of eyes, Pearson: Pearson Correlation Coefficient.

The mean angle κ was 277 μm ± 142 preoperatively and 217 μm ± 121 postoperatively in the overall group. The mean value of the hyperopic group was 307 μm ± 149 preoperatively and 238 μm ± 125 postoperatively while in the myopic group it was 208 μm ± 92 and 172 μm ± 94, respectively. This decrease in κ magnitude means that the line of sight was more nasal to the pupil center pre-operatively and still nasal but closer to the pupil center post-operatively. Fig 3 shows this shift in angle κ in horizontal and vertical axis (x and y direction), whereby the change in the mean horizontal axis was larger. This change in magnitude of κ was significant (p < 0.001; Fig 4).

### Correlation angle κ and CDVA

The correlation between angle κ and CDVA was analyzed pre- and postoperatively. There was no significant relationship in the overall group preoperatively (*p* = 0.979). The hyperopic group showed a significant correlation between κ and CDVA postoperatively but not preoperatively, even though κ was higher preoperatively. The myopic group did not show any correlation between κ and CDVA (Table 1).

**Table 1:** Correlation between angle κ and CDVA in all three groups, pre- and postoperatively (p-values).

	overall group	hyperopic group	myopic group
**preoperative**	0.979	0.238	0.948
**postoperative**	0.05	0.042	0.762

### Correlation angle κ and SI

The relationship between angle κ and SI pre- and post-operatively showed no significant differences in the subgroups or in the whole group. Thereby, all associations here were not statistically or clinically significant (Table 2).

**Table 2:** Correlation between angle κ and SI in all three groups, pre- and postoperatively (p-values).

	overall group	hyperopic group	myopic group
**preoperative**	0.336	0.550	0.184
**postoperative**	0.215	0.377	0.713

### Correlation angle κ magnitude change and SI or CDVA

**Fig 5:**
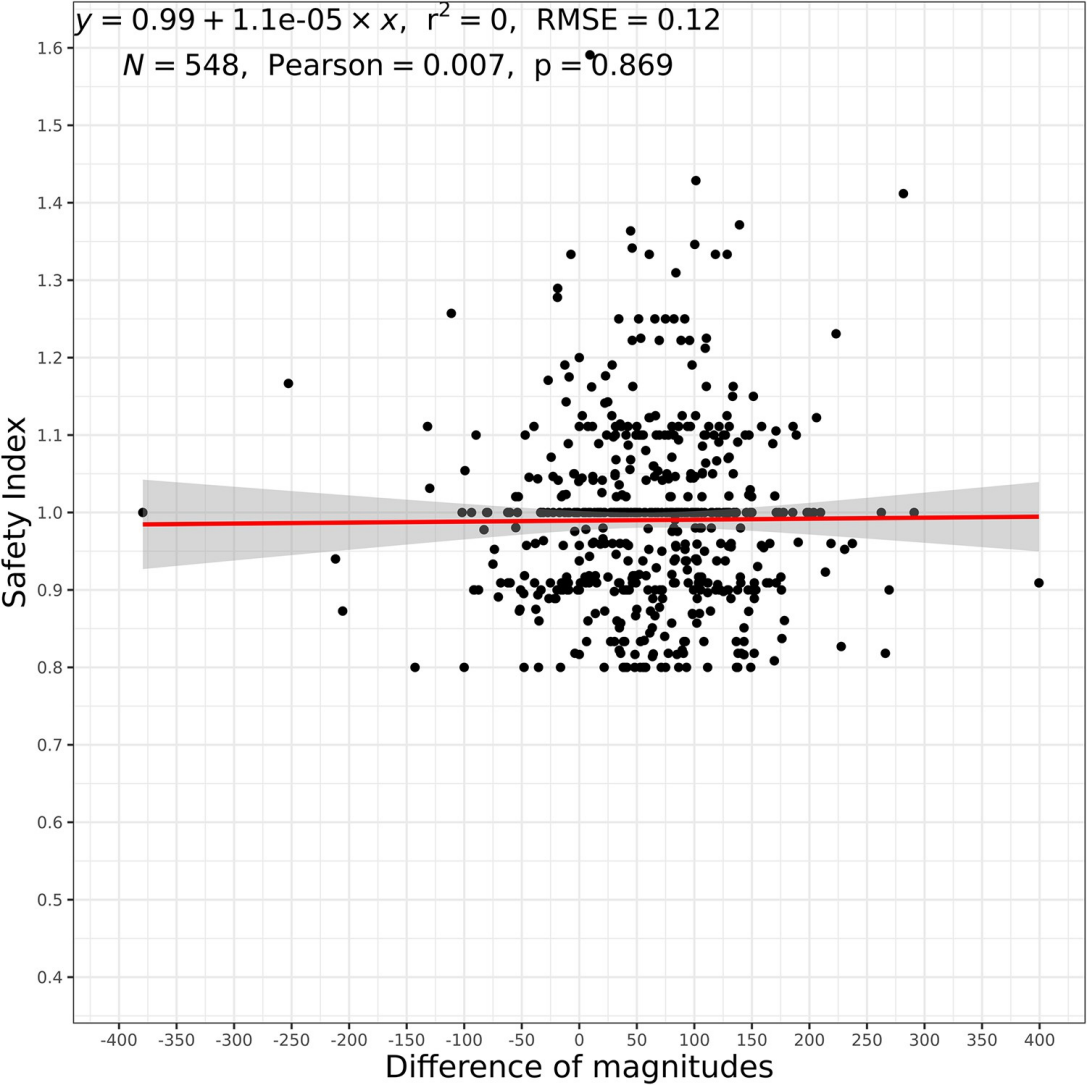
Difference in angle κ magnitudes in relation to SI (overall group). r^2^: coefficient of determination, RMSE: root mean squared error, N: number of eyes, Pearson: Pearson Correlation Coefficient.

**Fig 6:**
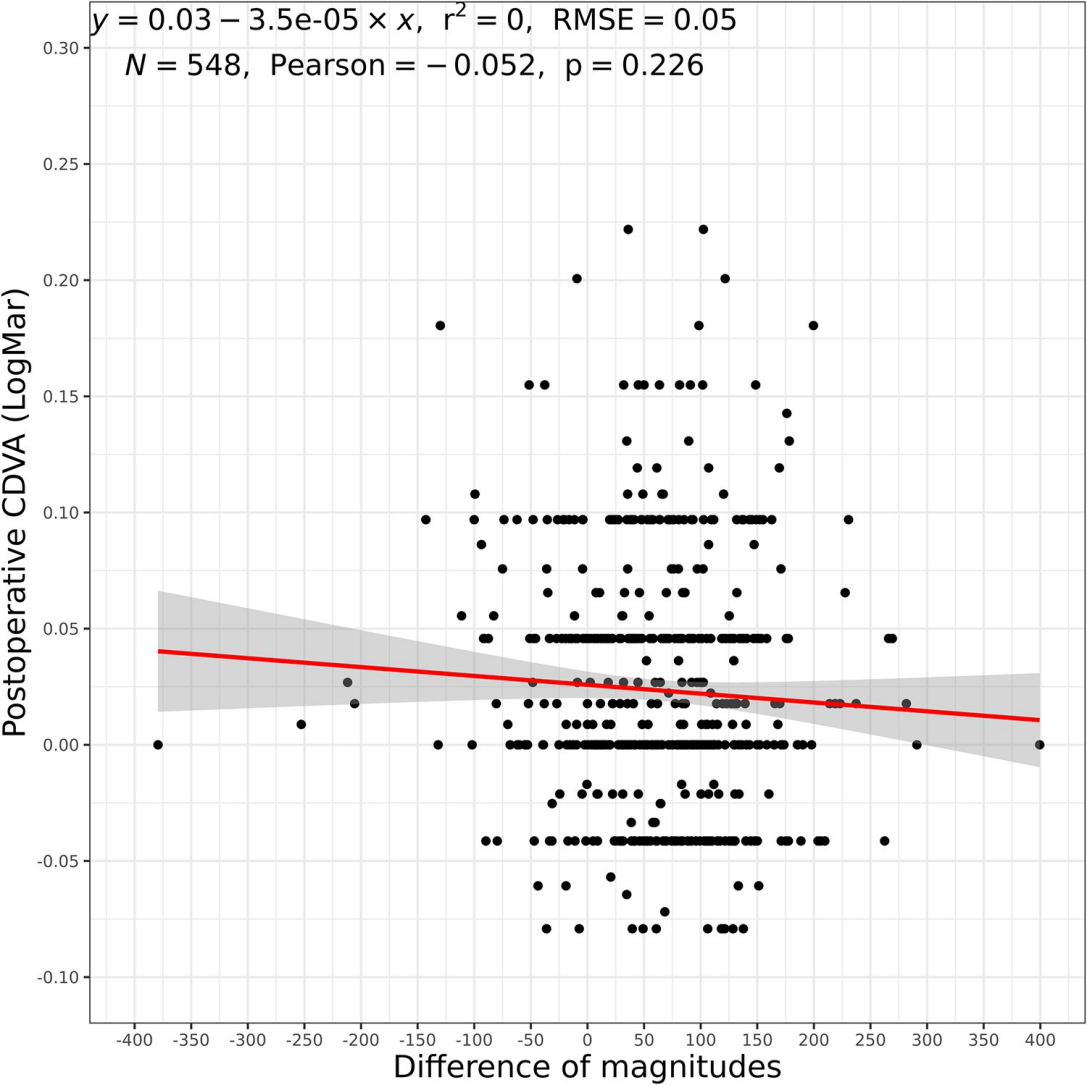
Difference in angle κ magnitudes in relation to CDVA (overall group). CDVA: corrected distance visual acuity, r^2^: coefficient of determination, RMSE: root mean squared error, N: number of eyes, Pearson: Pearson Correlation Coefficient.

Finally, the change of the angle κ magnitudes also showed no significant correlation to neither SI nor CDVA (p = 0.869 and p = 0.226, respectively, Figs 5 and 6). No significance was found in the subgroups either.

## Discussion

In the current study we first examined the distribution of angle κ magnitudes in eyes before a clear lens extraction. Next, we set out to investigate the change in angle κ magnitude after MIOL and LASIK surgery. Finally, we discussed the correlation of angle κ on the postoperative outcome after both procedures. Our study would be the largest among published studies of angle κ after MIOL.

A decrease of angle κ after phacoemulsification and MIOL implantation was presumed by *R*. *Wang et al*. and *Garzón et al*. [20, 21]. *S*. *Zarei-Ghanavati et al*. showed no change of angle κ after refractive surgery (PRK) [22] while our group showed in a previous study a change in its magnitude even intraoperatively during Excimer Laser vision correction compared to preoperative κ values [23]. Our expectation of a significant change in angle κ magnitude after MIOL and Bioptic procedure was confirmed by this study.

The analysis of angle κ distribution in an Iranian population investigated by *H*. *Hashemi et al*. showed that hyperopic eyes have a larger angle κ then myopic eyes and that emmetropic eyes hold the greatest values [24]. Others proved a similar occurrence [25, 26]. Factors associated with larger angle κ include shallower anterior chamber depth and older age [27]. Our study supports the findings of these studies and showed higher angle κ magnitudes in hyperopic and myopic eyes amounting to 99 μm preoperatively and 66 μm postoperatively.

The results of some studies suggest a correlation between large angle κ and unfavorable results occurring postoperatively [8]. Others could not find any significance of this suggested relationship. *Moshirfa et al*. summed up from previous studies that large angle κ could be a factor leading to a decentration of MIOL and consequently to vision dysphotopsias. A possible explanation is that with larger angle κ, central light rays would pass through the multifocal rings at the edge causing glare. But this correlation is not clearly proved [15, 28]. Our study does not analyze the centration of the MIOL compared to pupil center. Since the MIOL is centered on the circumference of the capsular bag one may suggest that higher distance between pupil center and line of sight may be related to discrepancy between capsular bag center and one of these anatomic centers. This question may be relevant to the performance of the MIOL but is beyond the scope of our current study.

*Karhanová et al*. showed in a more differentiated approach that at positive angle κ (pupillary axis is nasal compared to visual axis), temporal decentration of the MIOL has a worse effect regarding photic phenomena then does nasal decentration [15]. Some recommend considering angle κ when selecting patients for MIOL implantation and ruling out surgery, if applicable [11]. Angle κ larger than 400μm before MIOL implantation is related to more dysphotopsias, larger than 500μm with decreased visual quality [9, 29]. *H*. *Basmak et al*. also assume a negative effect of large angle κ on the postoperative result of laser treatment [26]. However, the data proving this relationship is very poor and partially demonstrated on an eye model only.

*C*. *Velasco-Barona et al*. and *Garzón et al*. could not find any significant association between angle κ and higher-order aberrations in their clinical trial. Furthermore, the visual acuity of the three different trifocal MIOLs does not seem to be affected by angle κ [10, 21]. *Cobo-Soriano* et al also excluded angle K as a negative factor influencing CDVA in bioptics patients, although they underwent corneal refractive surgery before lens replacement [30]. In our study, no negative effects were evident in CDVA or SI even in MIOL patients with high angle κ magnitudes after LASIK enhancement. Therefore, our findings go in line with previous data of *C*. *Velasco-Barona et al*. However, postoperative photic phenomena appear to have multifactorial causes (biometrical parameters such as shallow anterior chamber), making it difficult to identify a significant relationship to angle κ [15].

Centration of corneal ablation in laser refractive surgery has been widely discussed as it can affect the visual outcome. While centering over the pupillary center was previously assumed to be safe [31], more recent studies have prioritized the corneal light reflex (or visual axis) in this procedure [32–35]. This enhancement of safety and efficacy is more pronounced in hyperopic eyes because, as previously mentioned, they have a larger angle κ. This is negligible in myopic eyes since they commonly have small angle κ and therefore, a small distance between both points [36]. Accordingly, *M*. *Moshirfar et al*. recommend selecting the half distance between corneal light reflex and pupillary center [28]. Since angle κ changes intraoperatively, *Frings et al*. recommend centering the ablation based on the intraoperative measurements [23]. Moreover, the ametropia corrected in touch-up LASIK is normally very small and might be less sensitive to angle κ related to decentration of the excimer ablation.

Analyzing the role of angle κ in combination of clear lens extraction, MIOL implantation and a touch-up procedure (Bioptics) makes our study a unique research subject. According to the high variability of factors, our results show that at least angle κ does not need to be routinely evaluated in MIOL eyes receiving a laser enhancement surgery. Due to the current unclear published literature and disagreements, further studies are needed to support our findings.
